# Biotechnological Approaches to Lowering the Ethanol Yield during Wine Fermentation

**DOI:** 10.3390/biom11111569

**Published:** 2021-10-22

**Authors:** Ramon Gonzalez, Andrea M. Guindal, Jordi Tronchoni, Pilar Morales

**Affiliations:** 1 Instituto de Ciencias de la Vid y del Vino (CSIC, Gobierno de la Rioja, Universidad de La Rioja), 26007 La Rioja, Spain; rgonzalez@icvv.es (R.G.); andrea.martin@icvv.es (A.M.G.); 2 Faculty of Health Sciences, Valencian International University (VIU), 46002 Valencia, Spain; jtronchoni@universidadviu.com

**Keywords:** wine, low-alcohol, fermentation, global warming

## Abstract

One of the most prominent consequences of global climate warming for the wine industry is a clear increase of the sugar content in grapes, and thus the alcohol level in wines. Among the several approaches to address this important issue, this review focuses on biotechnological solutions, mostly relying on the selection and improvement of wine yeast strains for reduced ethanol yields. Other possibilities are also presented. Researchers are resorting to both *S. cerevisiae* and alternative wine yeast species for the lowering of alcohol yields. In addition to the use of selected strains under more or less standard fermentation conditions, aerobic fermentation is increasingly being explored for this purpose. Genetic improvement is also playing a role in the development of biotechnological tools to counter the increase in the wine alcohol levels. The use of recombinant wine yeasts is restricted to research, but its contribution to the advancement of the field is still relevant. Furthermore, genetic improvement by non-GMO approaches is providing some interesting results, and will probably result in the development of commercial yeast strains with a lower alcohol yield in the near future. The optimization of fermentation processes using natural isolates is, anyway, the most probable source of advancement in the short term for the production of wines with lower alcohol contents.

## 1. Introduction

Commercial wines have been experiencing a steady increase in alcohol levels since the 1980s. On average, the gain in alcohol has been about 1% (*v*/*v*) every ten years, currently reaching a total increase of 3–4% (*v*/*v*). There are two main reasons behind this trend. From one side, global climate warming affects, in different ways, the different physiological processes involved in grape berry ripening. Both sugar accumulation and phenolic and aromatic maturity develop faster, but the impact is greater for sugars. This imbalance prevents early harvesting from being an adequate solution to this problem, as it would lead to organoleptic defects associated with unripe berries. On the other hand, the consumer demand for tasty, structured, and full-bodied wines has pushed the market towards winemaking styles that require the harvest of very ripe grapes. As a result, quality grapes today tend to be richer in sugar content, leading to the observed increase in wine alcohol levels when all this sugar is fermented by yeasts [1].

Excess alcohol in wines is becoming a problem for the industry from several points of view. Wine balance is a delicate equilibrium between sweetness, acidity, bitterness, and aroma compounds, with alcohol as a vehicle for the perception of the latter. Ethanol surplus can lead to a sensory imbalance by promoting bitterness and masking fruit perceptions, and increasing the perception of sweetness, astringency and hotness. Besides this, sticking to moderate alcohol consumption becomes more difficult, and can therefore lead to increased risks for consumer health and road safety. Taxation based on alcohol levels in several importing countries is an additional incentive to look for wines with lower ethanol contents. Taken together, all of these factors might contribute to discourage wine consumption. Furthermore, from a bioprocess point of view, high levels of ethanol could lead to sluggish or stuck fermentation processes, with significant economic consequences.

In recent years, there has been a collective effort by researchers, engineers, and winemakers to develop approaches to limit the ethanol content of wines [2]. This community is targeting almost every stage of the production cycle, including, among other examples, grapevine clonal selection, vineyard management, winemaking practices adapted to unripe grapes, the use of yeast strains with a lower ethanol yield (often recombinant) or metabolic inhibitors, and partial dealcoholisation by physical means. Unfortunately, straightforward solutions, such as early harvesting or post-fermentation treatments, often have a negative impact on the wine quality (e.g., a green character and altered aromatic profile). Apart from sweet wines, most wines on the market are dry wines, which should contain very low levels of residual sugar. Solutions that result in less alcohol but an increase in residual sugars (i.e., stopping fermentation before completion) will drastically change the wine style, and will not be considered in this review. Here, we will present biotechnological solutions related to the fermentation process, mainly involving the selection and improvement of wine yeast strains for reduced ethanol yield, but also the use of some enzymes or metabolic inhibitors.

In this context, a pivotal concept is alcohol yield, which is the amount of ethanol produced per unit of sugar consumed. Most microbiological approaches to the reduction of the alcohol level in wines will target the ethanol yield of *S. cerevisiae* or alternative wine yeast species. Associated with the lowering of the alcohol yield is the concept of alternative carbon sinks (carbon-containing metabolic end products other than ethanol). Under standard fermentation conditions, about 60% of the hexose carbon consumed by *S. cerevisiae* ends up in the form of ethanol, 30% goes to carbon dioxide, and the remainder goes to biomass and less abundant metabolites. This led to the rule of thumb which states that for every 17 g/L of sugar in the grape must, an additional 1% *v*/*v* of alcohol wine is to be expected in the wine. Reducing the alcohol yield requires the diversion of the metabolic flux from ethanol to alternative carbon sinks.

However, alcoholic fermentation is the metabolic hallmark of *Saccharomyces cerevisiae*, the yeast species dominating most wine fermentation processes in the industry, either spontaneously or because it is used as a starter culture. This species always shows a high ethanol yield during fermentation, with very little biological diversity [3,4]. As an example, a survey of 72 *S. cerevisiae* strains from various geographical origins and ecological niches found great phenotypic diversity for other traits, but almost no differences in ethanol yield [4]. Below, we describe the different efforts made to overcome this biotechnological hurdle.

## 2. Genetic Improvement of *S. cerevisiae*

### 2.1. Genetic Engineering

**Figure 1:**
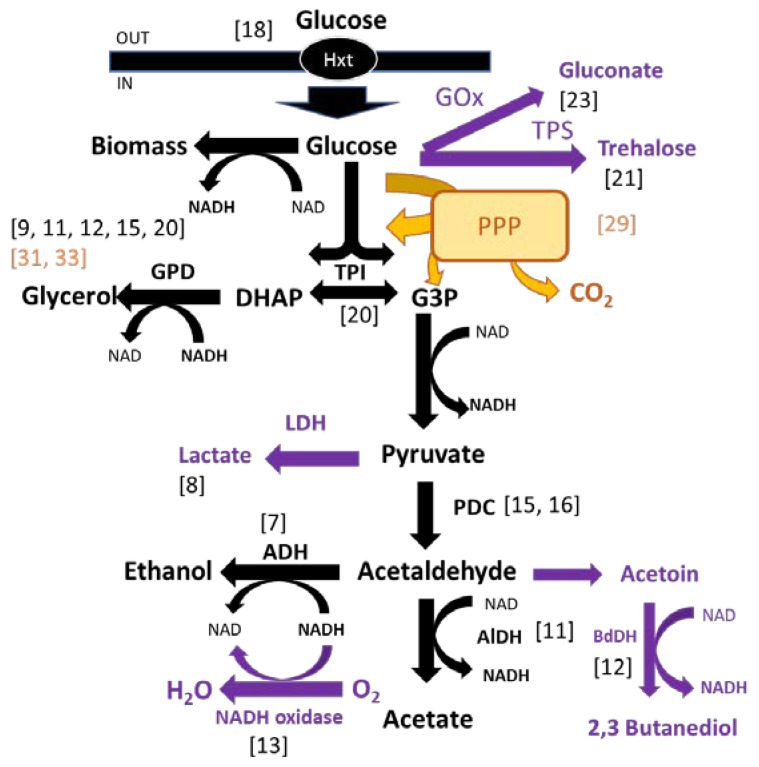
Diagram of the alcoholic fermentation pathway with indications of the steps affected by the different genetic modifications assayed to lower ethanol yields. References in black: genetic engineering. References in yellow: adaptive evolution. Metabolites outside of the central ethanol fermentation pathway are indicated in purple.

Since the 1990s, it has been a constant in wine biotechnology that product improvement and problem solving tend to be addressed, in the first instance, by the genetic engineering of wine yeasts. However, the wine industry is not the ideal breeding ground for genetically modified organisms due to restrictions to GMOs on food production in most countries, and their negative perception by wine consumers [5]. The only two recombinant wine yeasts that have been commercialised so far do not seem to have become bestsellers in the markets they were introduced in [5,6]. Therefore, none of the recombinant strains described in this section were intended for direct commercialization. Nevertheless, their study provided useful information for the improvement of yeast strains by alternative approaches. Indeed, in several instances, the reduction in alcohol levels appeared as a side-effect of genetic manipulations which were not intended for that purpose. As mentioned above, when it comes to reducing the alcohol level, a key issue to guide genetic engineering is the choice of carbon sinks. Figure 1 shows a summary of the main enzymatic reactions involved in alcoholic fermentation, including those referred to on the following lines.

Alcohol dehydrogenase (ADH) catalyses the final step in the production of ethanol during alcoholic fermentation. It is therefore a rational deletion target for limiting ethanol production. However, cell metabolism is complex and depends on a set of biochemical reactions that must be balanced in several ways, especially regarding the redox balance. For this reason, simple logics often lead to unwanted results. For example, alcohol dehydrogenase deletion leads to a reduction in ethanol production and an increase in that of glycerol, but these recombinant strains are unable to grow under anaerobic conditions [7]. This genetic modification also results in higher levels of acetic acid and acetaldehyde, far beyond levels suitable for commercial wines.

In addition to the modulation of the expression of endogenous genes by shutting down or upregulating their expression, genetic engineering allows for the heterologous expression of genes from other biological species. In 1994, Dequin and Barre [8] expressed lactate dehydrogenase from *Lactobacillus casei* in *S. cerevisiae* to divert some of the pyruvate formed in glycolysis to lactate formation. The engineered yeast strain simultaneously performed alcoholic and lactic fermentations. The redox balance was not affected, as the formation of lactic acid is compensated by the disappearance of ethanol in equimolecular amounts, for the same consumption of NADH. Thus, a reduction of 1% ethanol leads to an increase in 15 g/L lactic acid. The increased lactate content of these wines contributed to a higher total acidity. This feature could be useful to compensate for low acidity, which is a problem affecting some grape varieties and growing regions, and is also related to global warming. However, this also sets a limit to this approach, as excessive acidity would make the wines unpalatable.

Glycerol contributes positively to the viscosity, body, and sweetness of wine. It has been a preferred carbon sink for the genetic engineering of wine yeasts. The overexpression of *GPD1*, coding for glycerol 3-*P* dehydrogenase, results in a significant increase in glycerol (plus 28 g/L) and a reduction in ethanol (minus 15 g/L), and also in the appearance of excess acetate and acetoin to restore the intracellular redox balance during fermentation [9]. Researchers identified Ald6 as the aldehyde dehydrogenase isoenzyme responsible for most of the acetate production of *S. cerevisiae* [10]. However, the knockout of *ALD6* resulted in an additional increase in acetoin production which, at high concentrations, negatively affects wine aroma, providing buttery notes [11]. Following this line of research, the problem of acetoin overproduction was tackled by overexpressing *BDH1*, which codes for butanediol dehydrogenase [12]. This enzyme catalyses the conversion of acetoin to 2,3-butanediol, a compound with no apparent sensory impact. Using this recombinant strain, wines show a significant reduction in ethanol, and above 26 g/L of glycerol content. The number of different genetic modifications required to achieve this objective is in itself an indication of the difficulty of using this as a general approach to developing wine yeast strains with a low ethanol yield.

The different adjustments that were required to develop the above-described recombinant yeasts respond to metabolic constraints concerning the redox balance and the availability of an adequate pool of cofactors. Therefore, some authors have tried to act directly on the reactions of the redox system. By cloning an NADH oxidase for the regeneration of the reducing power, and with controlled oxygenation, not only is a decrease in ethanol obtained but also a significant increase in the levels of acetaldehyde, acetic acid, and acetoin [13].

There are two enzymatic steps from pyruvate to ethanol during alcoholic fermentation (Figure 1). The final one catalysed by alcohol dehydrogenase has been discussed above. The first, from pyruvate to acetaldehyde, catalysed by pyruvate decarboxylase (PDC), was also taken as an improvement target by some researchers. In the absence of PDC activity, the Crabtree effect is abolished, but this activity is required for acetyl-CoA synthesis in the cytoplasm and NAD^+^ regeneration. It is therefore essential for *S. cerevisiae* growing on glucose [14]. The deletion of *PDC1* resulted in a fourfold reduction of the PDC activity, with an increase in pyruvate levels, but no reduction of ethanol levels [10]. The deletion of *PDC2*, coding for a transcription factor involved in PDC gene expression, results in 19% PDC activity, with a clear reduction in the ethanol content, combined with an increase in glycerol and pyruvate release [15]. This effect is enhanced by increased glycerol-3-phosphate dehydrogenase activity, through the overexpression of *GPD1*, without the over-production of acetic acid and acetaldehyde associated with *GPD1* over-expression in the original strain. Cuello et al. [16] replaced one of the native *PDC2* alleles in different diploid wine yeast strains by a truncated *PDC2* allele. These engineered strains showed a 2% ABV reduction without any increase in acetate production. An alternative way to produce cytosolic acetyl CoA in *pdc^-^* strains was developed by substituting Acs1 and Acs2 (acetyl coA synthetase) with a heterologous pyruvate carboxylase and a transacetylase [17]. This strain produces glycerol, succinate, pyruvate and acetate from glucose, but not ethanol. The additional deletion of gpp1 and gpp2 (glycerol 3p phosphatase) avoids the production of these metabolites, including glycerol, and increases the growth rate.

Crabtree-negative strains of *S. cerevisiae* were also constructed by substituting all of the active hexose transport genes with a single chimeric one [18]. This strain does not produce ethanol as long as oxygen is available for respiration. However, its growth kinetics are extremely slow. Further possibilities of using the respiratory metabolism of yeast to reduce the ethanol yield during wine fermentation will be examined below in a specific section.

Triose-phosphate isomerase, encoded by *TPI1*, is the enzyme catalysing the interconversion between the two products resulting from the breakdown of fructose 1,6-bisphosphate: dihydroxyacetone phosphate and glyceraldehyde 3-phosphate (Figure 1). The topology of the pathway suggests that *TPI1* deletion would result in equimolar levels of glycerol and ethanol precursors. However, in practice, such deletion strains are unable to grow on glucose due to a redox imbalance [19]. In order to improve industrial glycerol yields, additional genetic modifications have been introduced in strains deleted for *TPI1* [20]. These authors deleted *ADH1* (coding for alcohol dehydrogenase) and *TPI1* in a yeast strain while overexpressing *GPD1* (coding for glycerol-3-phosphate dehydrogenase), *FPS1* (coding for a glycerol transporter) and *ALD3* (coding for an aldehyde dehydrogenase). These strains produce very low levels of ethanol (about 25% of the parent strain), and their glycerol levels are multiplied by 30. However, their growth rates were strongly reduced.

Another alternative carbon sink is yeast biomass. Despite the portion of carbon going to biomass being quantitatively small during wine fermentation, some authors have targeted reserve sugars to divert the carbon flux from ethanol. A moderate overexpression of *TPS1*, the gene coding for trehalose-6-phosphate synthase, involved in trehalose biosynthesis, resulted in a clear reduction in the ethanol yield, without an apparent impact on the redox balance, according to the profile of the byproducts analysed [21].

Global transcription machinery engineering (gTME) technology has recently been employed to construct a low-alcohol yeast strain. One strain with a reduced ethanol yield was selected from a library of mutants in *SPT15* (coding for a transcription factor) generated by random directed mutagenesis [22]. The pleiotropic effect of this mutation downregulates hexose transport and ethanol production, and upregulates glycerol production. Acetic acid is not increased, but the mutant shows slower growth kinetics than the parent strain.

The treatment of must with glucose oxidase is an alternative method to decrease the alcohol content of wine that will be discussed in the corresponding section. Based on this method, Malherbe et al. [23] cloned a glucose oxidase gene from *Aspergillus niger* in a laboratory strain of *S. cerevisiae*. Wines produced with the recombinant strain contained 2% less ethanol. The recombinant strain showed antimicrobial activity against acetic and lactic acid bacteria, probably due to the H_2_O_2_ released by the oxidation reaction.

The CRISPR/Cas9 (Clustered Regularly Interspaced Short Palindromic Repeats/CRISPR associated protein 9) genetic modification system has already been implemented in *S. cerevisiae*, including wine yeasts [24,25]. The basic CRISPR/Cas9 system consists of a guide RNA molecule (gRNA) and an endonuclease (Cas9). The endonuclease mediates a double strand break guided by the hybridization of gRNA with genomic DNA. This triggers the DNA repair mechanisms of the host cell. The system can be designed to target the nuclease to the gene of interest, and to perform more or less large insertions and deletions. An advantage of this system is that, unlike other genetic engineering techniques, it can easily be tuned to leave no trace of the ancillary genetic material. In addition, it is less dependent on traditional transformation markers, and makes easier the manipulation of diploid loci. Nonetheless, microorganisms obtained with this system still fall under European Union GMO regulations. The genes *GPD1* and *ATF1* have been cloned in a haploid wine strain using this technique [26], with no differences in ethanol production between the strains, contrary to the results obtained in previous works [9,15]. Other works applying this technique for the reduction of wine alcohol content will certainly appear soon.

### 2.2. Random Mutagenesis

Genetic improvement by random mutagenesis involves the artificial increase in mutation rates by treatment with physical or chemical agents (UV radiation, X-rays, ethyl methane sulfonate, nitrosoguanidine) coupled with a suitable selection strategy [27]. To be effective, the intensity of the treatments must be lethal for most of the cells in the population. However, given the random nature of the process, the number of cells that must remain viable in order to capture the expected mutations is often still too high for strain-by-strain analysis. It is then critical to establish appropriate selection criteria that link selectable or detectable phenotypes to technologically relevant features of the mutant strains. While this seems like an easy task for some improvement outcomes—for example, those related to the robustness of yeast strains—improvements in wine quality, including the reduction of alcohol levels, require indirect and non-obvious selection criteria.

An improved strain of *S. bayanus* was developed by random mutagenesis, and was marketed as a low ethanol producer (Oenoferm^®^ LA-HOG from Erbslöh, Geisenheim, Germany). According to commercial information, the genetic improvement process involved two stages of chemical mutagenesis. The first one, with selection under hypersaline conditions, was intended to recover strains with an improved HOG (High Osmolarity Glycerol) response. In the second, the selection pressure was established with pyrazole, an inhibitor of alcohol dehydrogenase. The commercial strain was selected among the 40 strains recovered from this last step. During the fermentation of the Pinot noir grape juice, this strain produced 12.9 g/L glycerol and 94.6 g/L ethanol, compared to the 6.4 g/L and 102.4 g/L, respectively, produced by the parent strain.

### 2.3. Adaptive Laboratory Evolution

Adaptive laboratory evolution (also known as experimental evolution) consists of recreating in the laboratory, in an accelerated way, the natural selection process, promoting the selection of the mutations that are most useful to our purpose. This technique shares several features with random mutagenesis [27], including some limitations, such as that it cannot be easily targeted to specific genes, that dominant mutations are favoured, and the difficulties of connecting readily selectable phenotypes to technological traits. However, it also offers advantages, as the strains obtained in this way do not fall under GMO regulations. In this case, mutations appear spontaneously in the population during each round of duplication (although it can eventually be combined with random mutagenesis). Yeasts are grown in a selective environment in which the desired metabolic changes would favour their growth. Once such a mutation appears, and after several generations under the same selection conditions, many individuals in the population will harbour the desired mutation. In addition, new variants can arise from the original one, and in this way the favourable mutations for the intended technological objective can be accumulated in the evolving lineage. The random nature of spontaneous mutations, and the fact that many of them can be added over time during experimental evolution, gives some advantages to this approach over genetic engineering. As indicated above, the challenge of this technology is to anticipate the metabolic pathways to be altered in order to reach the expected technological output, and the growth conditions that will give advantage to the yeast cells improved in that way.

In the context of alcohol level reduction, adaptive laboratory evolution has been used to channel carbon flux towards the pentose phosphate pathway (PPP; Figure 1). The selective pressure was established using gluconate as the only carbon source [28]. Gluconate is a substrate poorly assimilated by *S. cerevisiae* and metabolized by the PPP. The rationale for targeting PPP was that, compared to glycolysis, an additional CO_2_ molecule is released for each glucose molecule entering the pathway (meaning that this carbon will not contribute to ethanol production). The selected strain showed a 1.5-fold increase in flux through the PPP compared to the ancestral strain, corresponding to a reduction of 2 g/L in ethanol levels. A fivefold increase in flux through the PPP would have been necessary to reduce the ethanol level of wine by approximately 1% (vol/vol). However, selected evolved strains did not show a reduced ethanol yield, but an increased production of esters [29], which can be useful, but not for the original purpose.

Other authors have used experimental evolution to push carbon flux towards glycerol production. The process was based on a long-standing strategy for the industrial production of glycerol based on sulphite addition [30]. The reaction between sulphite and acetaldehyde decreases the availability of acetaldehyde for ethanol production and NADH oxidation, and cells must compensate the redox balance by increasing glycerol production. Kutyna et al. [31] used this principle to drive the evolution of a laboratory strain, with sodium sulphite as a selective agent, at an alkaline pH. Strains evolved in this way did show a relative increase in glycerol yields, but there was a very limited impact on ethanol yield. In addition, genetic analysis showed that the sulphite tolerance and glycerol yield were not linked in the evolved strains.

Glycerol is the major osmolyte in yeasts [32]. Tilloy et al. [33] targeted glycerol production to reduce the ethanol yield from a wine yeast strain. In this case, the selective pressure was osmotic stress and the HOG intracellular signal transduction pathway. Strains evolved in a hypersaline medium were selected and further improved by sporulation and back-crossing. The resulting strains showed reduced ethanol yield, increased glycerol yield, and did not produce acetic acid. However, the metabolic analysis revealed that, contrary to expectations, the overproduction of glycerol was not due to the activation of the HOG pathway. Compared to recombinant strategies which were also based on glycerol overproduction, the authors found the evolved strains did not show the drawbacks of *GPD1*-overexpressing strains. At the pilot scale, these strains produced wines with 0.4% (*v*/*v*) to 1.3% (*v*/*v*) less alcohol than the control (for Syrah and Grenache grapes, respectively). Grenache wines were tasted, and no organoleptic defects were detected. This strain has been commercialized.

## 3. Alternative Wine Yeast Species

Microbial ecology studies of grape juice and wine fermentation clearly indicate that, most often, *S. cerevisiae* is a minor species at the beginning of the fermentation process. In contrast, it is almost invariably the dominant one at the end of spontaneous fermentations. Therefore, this species is considered particularly well adapted to the process, and has been selected for use in inoculated fermentations. The other yeast species present during the initial stages of fermentation were traditionally considered to be spoilage microorganisms, and therefore undesirable. Over time, after inoculation with *S. cerevisiae* became common practice, non-*Saccharomyces* wine yeast species have been found to harbour interesting characteristics, such as their contribution to the aroma and complexity of the wine, and their ability as microbiological control agents [34]. Several commercial strains of non-*Saccharomyces* yeast are currently available for use in wine fermentation, including *Torulaspora delbrueckii*, *Lachancea thermotolerans* and *Metschnikowia pulcherrima,* among the most common yeast species [35]. In addition to their features related to the sensorial quality of wines, one potentially interesting feature of some non-*Saccharomyces* species is their lower ethanol yield compared to *S. cerevisiae*. However, only during the last decade has the possibility of using alternative yeast species to reduce alcohol content been considered [36]. A summary of the results described below, using different non-*Saccharomyces* yeast species to obtain lower alcohol wines, is shown in Table 1.

The cryophilic species *Saccharomyces uvarum* has been described as a low ethanol, low acetate, and high glycerol producer yeast [37,59,60]. In experimental fermentations, the ethanol contents of wines produced with two strains of *S. uvarum* were 0.5 and 0.3% ABV lower than *S. cerevisiae* wine, and their hybrids showed ethanol contents which were intermediate between the parent species [37]. An important reduction (1.7% ABV) was obtained with a strain of *S uvarum* in Merlot wine, but it showed predominantly negative attributes in a sensory analysis, with aromas of meat and barnyard [38]. Another cryophilic species, *Saccharomyces kudriavzevii*, has been described as a high glycerol producer [61]. Although *S. kudriavzevii* is not found in winemaking, *S. cerevisiae* × *S. kudriavzevii* hybrids are often isolated from cold-climate wine environments. However, the ethanol yield of these hybrids has been found to be higher than that of *S. cerevisiae* [62].

In 2011, Magyar and Toth [63] studied the ethanol yield of several strains of *S. cerevisiae*, *S. uvarum/bayanus*, *Candida zemplinina* (currently *Starmerella bacillaris*) and *Candida stellata*. All of the other species showed a lower ethanol yield than *S. cerevisiae*, in particular *C. zemplinina* isolates. In addition, this species is fructophilic and shows a high glycerol yield. A great number of strains of this species have been explored for ethanol production [64,65]. Wines made with the selected strains contained, at best, 0.6% ABV less than the control wine [46,47].

Gobbi et al. (2014) [66] assessed the ethanol yield of 32 strains belonging to 8 different species, compared to a control strain of *S. cerevisiae*, and found that the species *Hanseniaspora uvarum* showed the lowest yield, followed by *Zygosaccharomyces sapae* and *Zygosaccharomyces bisporus*. The authors highlight the variability among strains of the same species, and the correlation found between ethanol yield and secondary metabolite production. Mestre et al. (2017) [67] also selected a strain of *H. uvarum* among 117 strains belonging to 32 different species. Malbec wine produced with the selected strain [50] had a 0.9% drop in ethanol in compared to that made from *S. cerevisiae*, and a good qualification in a sensory analysis.

Contreras et al. (2014) [40] studied the ethanol yield and sugar consumption of 50 yeast strains, with the aim of selecting suitable strains to lower the alcohol content of wine. The authors highlighted the potential of strains of the species *C. stellata*, *Schizosaccharomyces malidevorans* and, mainly, *M. pulcherrima*. A selected strain of *M. pulcherrima* was used for sequential fermentations with *S. cerevisiae,* and allowed for an ethanol reduction of 1.6% (*v*/*v*) for Shiraz must and 0.9% (*v*/*v*) for Chardonnay must. An increase in ethyl acetate and 2-methyl propanol was observed, which can negatively impact the wine quality. More recently, they fermented Merlot must with *M. pulcherrima* in a sequential inoculation with *S. cerevisiae* [38]. Compared to wine inoculated with *S. cerevisiae*, wine from spontaneous fermentation contained 0.7% (*v*/*v*) less ethanol, and wine fermented by *M. pulcherrima* contained 1% (*v*/*v*) less. In the sensory analysis, spontaneously fermented and *M. pulcherrima* wines were indistinguishable, and very similar to the control. Coinoculation with *M. pulcherrima* and *S. uvarum*, in sequential inoculation with *S. cerevisiae*, allowed a stronger reduction (1.7% *v*/*v*) than using these strains separately (0.8–1.0% *v*/*v*) [39].

Rossouw and Bauer (2016) [49] studied the ethanol yield of 91 yeast strains, including some isolates of *S. cerevisiae* and two commercial strains as control strains. The authors highlight the variability in the rate and extent of sugar utilisation between strains of the same species, except for *S. cerevisiae* species, and the differences in aroma compounds produced by strains affecting the wine aroma. Wines made with selected strains of *Hanseniaspora opuntiae*, *Hanseniaspora uvarum*, and *Pichia kudriavzevii*, in sequential inoculation with *S. cerevisiae*, contained between 0.4 and 1.5% ABV less than the control wines.

There are currently several strains of *T. delbrueckii* for winemaking in the market which have been selected for their contribution to the aromatic complexity of wine. Regarding the ability to lower the alcohol content, there was a 0.5% *v/v* reduction in the ethanol content compared to the *S. cerevisiae* control wine [52]. Puškaš et al. (2019) [53] studied the effectiveness of one commercial strain of *T. delbrueckii* and one strain of *M. pulcherrima*, in co-inoculation with *S. cerevisiae* and/or *S. bayanus*. In pasteurised must, a reduction of 0.9% (*v*/*v*) in ethanol concentration was achieved with *Metschnikowia* and 0.5% with *Torulaspora*, but this effect was lost in natural must, probably due to the low dominance of the selected strains.

*L. thermotolerans* has also been introduced in the market because its positive effects on wine aroma, total acidity, and volatile acidity [68]. Wines obtained with the sequential inoculation of *L. thermotolerans* and *S. cerevisiae* contained 1.2% less ethanol than the control wine, but suffered a decrement in aroma quality [55].

Some authors are also exploring yeast cell immobilization to improve effectiveness and process control (Table 1). Canonico et al. (2016) [56] studied the use of immobilised alternative yeasts, together with free *S. cerevisiae* in sequential inoculation. Using *Starmerella bombicola*, *H. uvarum*, *Hanseniaspora osmophila* and *M. pulcherrima*, ethanol reduction values from 1.0 to 1.6% (*v*/*v*) were achieved. Changes in the volatile compounds were also observed. It was predicted that these could potentially affect the sensory properties of the wines, either positively or negatively, depending on the case, but the study did not include a sensory evaluation. Finally, the authors indicated that this technology makes the process more expensive. Domizio et al. (2018) [58] obtained a 1.7–2.4% reduction of ethanol levels with immobilized cells of *Schizosaccharomyces japonicus* in co-inoculation and sequential inoculation with free *S. cerevisiae*. The levels of acetoin, ethyl acetate, and acetate esters were higher in both wines, and acetic acid and glycerol were higher in wine made by sequential inoculation.

Several of the studies described above show some promise for non-*Saccharomyces* yeast used in sequential or co-inoculation with *S. cerevisiae* for the reduction of ethanol yields during wine fermentation, and hence the alcohol content of wines. However, even under laboratory controlled experimental conditions, the reduction values were generally moderate (Table 1), and difficulties associated to scale-up were often reported. Fairly different reduction values are published by different laboratories, which is not surprising because the authors did not follow standardized conditions (the optimization of fermentation conditions is usually a secondary objective of their work), and different strains were used in each case. According to the available information, when developing protocols aiming at alcohol level reduction, attention should also be paid to the impact of strains and fermentation conditions on the sensory properties of the wines. In addition, the use of non-*Saccharomyces* species for alcohol reduction faces technological challenges common to other oenological applications of these strains. These include difficulties for the industrial production of most of them as active dry yeast, a generally low tolerance to sulphiting agents, low imposition, or considerations about compatibility with *S. cerevisiae* starters (which will almost always be necessary, in sequential or simultaneous inoculation with non-*Saccharomyces*, to ensure that fermentation is completed).

## 4. Aerobic Fermentation

### 4.1. Non-Saccharomyces Species

Glycerol has been the target in many attempts to reduce ethanol yields by genetic improvement (see above). However, reducing the alcohol content by 1% (*v*/*v*), by diverting all excess carbon to glycerol, can result in more than 15 g/L extra glycerol in the wine. This sets a technological limit to this strategy, as a further increase in the glycerol yield would lead to values well above the commercially acceptable levels. However, almost every alternative carbon sink can have a detrimental sensory impact if produced in excess, even for compounds with positive sensory attributes.

In this context, some authors, including our research group [69,70], proposed CO_2_ generated by the aerobic respiration of yeast as a neutral carbon sink. By respiration, all of the carbon present in sugar is converted to CO_2_, in a process that requires molecular oxygen. However, *S. cerevisiae* is a Crabtree-positive yeast. This implies that, except under very small concentrations of glucose, it will mostly ferment sugars and produce ethanol, even in the presence of oxygen. Therefore, the proposal was made to perform a multi-starter fermentation with *S. cerevisiae* and alternative yeasts with higher respiration capacities [70]. The appropriate yeast would be inoculated into the must, and during the first hours, oxygen would be provided to the fermentation tank. After some time, the forced aeration would be stopped, and the traditional process would follow. *S. cerevisiae* could be inoculated from the beginning (co-inoculation), or when the aeration is stopped (sequential inoculation). The more sugar is respired during the aeration process, the lower the ethanol yield of the overall process will be.

The availability of suitable yeast strains was a prerequisite to reach this goal. To be useful, these strains must be able to respire in the stressful conditions of grape must, with no negative impact on the wine sensory properties. In a screening of 65 strains from 28 yeast species, Quirós et al. (2014) [71] found an interesting result for some *S. cerevisiae* strains. Despite the Crabtree effect, the residual respiration activity of some of them was enough to allow for a substantial reduction in the ethanol yield according to the measured respiration quotient (RQ) values. However, *S. cerevisiae* strains showed a boost in their acetic acid production when provided with air. In contrast, several non-*Saccharomyces* strains showed a combination of metabolic features suggesting that they might be suited for use in the reduction of alcohol levels. Namely, some strains showed a moderate-to-high sugar consumption rate, very low acetic acid production, and a low RQ (which means a low expected ethanol yield). Indeed, one strain of *M. pulcherrima* was used in a co-inoculation with *S. cerevisiae* in laboratory-scale bioreactors for the fermentation of natural grape must (260 g/L sugar content). Several inoculation and aeration rates were assayed. The alcohol level reduction reached 3.6% (*v*/*v*) with a somehow too-high volatile acidity, or 2.2% (*v*/*v*) with acceptable volatile acidity levels [43]. Some environmental factors that can be controlled during wine fermentation—such as nutrients, temperature, and dissolved oxygen levels—will certainly affect both the ethanol and acetic acid yield by different yeast strains. Interestingly, strains of *M. pulcherrima* and *Candida sake* show low acetic acid production almost independently of the fermentation conditions [72].

Fermentation conditions leading to lower ethanol yields based on the use of non-*Saccharomyces* strains under aerobic conditions have been studied by these and other authors, using *T. delbrueckii*, *M. pulcherrima*, *Zygosaccharomyces bailii*, *C. zemplinina*, *M. pulcherrima*, *Pichia guilliermondii, Pichia kluyveri*, *Candida oleophila*, *Starmerella bombicola*, or *Hanseniaspora vineae* [44,45,48,51,57,73,74]. The authors have worked with different aeration times and air flows, free or immobilized cells, and different varieties of white grapes (Table 1). As a trend, increased aeration leads to better results in terms of reduced alcohol levels, but also to a greater impact on the volatile compounds (including volatile acidity) and a lower rating from sensory panels. It can be concluded that the results are promising, but the process needs to be further developed, including the criteria for the selection of non-*Saccharomyces* strains for this application, in order to develop useful protocols for the industry.

### 4.2. S. cerevisiae Strains Improved for Aerobic Fermentation

As mentioned above, one major drawback for the use of *S. cerevisiae* under the aerobic growth conditions required for respiration was that strains of this species produced unacceptable acetic acid levels under these conditions. In order to overcome this problem, the researchers turned to other wine yeast species. Although this strategy is yielding some promising results (see the previous section), and though the use of non-*Saccharomyces* species could bring some other benefits to the wine, it also hinders the fermentation management in the winery. The use of a single yeast starter that will at the same time be able to reduce alcohol levels, and drive fermentation to the end, while not increasing volatile acidity, would be an optimal alternative.

A wine strain of *S. cerevisiae* improved for lower acetic acid production was expected to be able to meet all of these requirements. Indeed, recombinant wine yeast strains partly defective in carbon catabolite repression (CCR) due to *REG1* gene knockout were shown to produce reduced levels of acetic acid under anaerobic conditions [75]. Interestingly, *reg1* mutant strains can grow on non-preferred carbon sources (glycerol or galactose, for example) in the presence of the non-metabolizable glucose analogue 2-deoxyglucose (2DG). In contrast, strains with an intact CCR will be inhibited under these growth conditions. Although this link between CCR and acetic acid production under aerobic conditions is not yet clarified, it is possible to take a practical advantage of it to design experimental evolution strategies to develop *S. cerevisiae* wine yeast strains which are better suited for alcohol level reduction. This was recently performed by Guindal et al. [76] using galactose as a carbon source in the presence of growing amounts of 2DG for around 100 generations. Several evolved strains from different wine yeast genetic backgrounds were indeed derepressed for glucose, and had been indirectly selected for lower acetic acid production under aerobic conditions. As a trend, the glycerol yields were also increased for the selected evolved strains. However, some strains had to be discarded due to impaired fermentation kinetics. Indeed, there is always a risk of unwanted change during adaptive laboratory evolution. This must be managed by the careful design of experimental conditions and a thorough characterization of the derived strains before they can by promoted to industrial applications.

On the other side, in the framework of the European project CoolWine (ERA CoBioTech), researchers are working on the development of rational strategies for adaptive laboratory evolution aiming at alcohol level reduction. For *S. cerevisiae,* the aim is to develop new strategies to reduce the above-mentioned problem of excess acetic acid production in the presence of oxygen, while for non-*Saccharomyces* strains, the aim is to improve their survival and competitiveness in the context of winemaking.

Although the general trend for *S. cerevisiae* strains is the production of high volatile acidity under aerated wine fermentation conditions, we recently found rare isolates showing acceptable acetic acid yields under aerobic conditions [77]. These strains show promise to be used for alcohol level reduction by sugar respiration, without resourcing to non-*Saccharomyces* yeast or the requirement for genetic improvement. However, genetic improvement will still be necessary to ensure enough genetic diversity for industrial applications (a single strain cannot fulfil all industry requirements), and to take advantage of the features of commercial strains that have previously shown optimal features for winemaking.

## 5. Enzymatic Treatment of Grape Must

One conceptually simple approach for the reduction of the ethanol content of wines is to remove excess sugar from the grape juice before fermentation. Biotechnologically, this goal can be achieved using enzymes, and the enzymatic activity proposed for this application is glucose oxidase. This enzyme catalyses the oxidation of glucose to gluconic acid. The reaction involves molecular oxygen as a co-substrate, and releases hydrogen peroxide as a by-product.

The use of glucose oxidase during the pre-fermentation stages to produce low-alcohol wines was first proposed by Villetaz (1986) [78], and was optimised by this and other authors (as reviewed in [79]). However, the use of glucose oxidase is not among the procedures included in the International Code of Oenological Practices [80].

The procedure proposed by Pickering et al. [81] involved an initial grape must deacidification to accommodate the pH to that which is optimal for the enzyme used, as well as aeration during the enzymatic treatment. Catalase was added with glucose oxidase to remove the hydrogen peroxide formed in the reaction. The removal of the hydrogen peroxide improves the yeast survival and the activity of glucose oxidase enzyme itself. By treating Riesling grape juice for 10 h under these conditions, these authors produced wines with an alcohol content reduced by 3.7% (*v*/*v*) [82]. However, gluconic acid negatively affected the taste of the wine, fruity aromas were perceived with less intensity, and their persistence was also reduced [83]. A second deacidification or a sweetening were proposed to alleviate the effect of gluconic acid [81].

Röcker et al. [84] assayed several methods of deacidification following glucose oxidase treatment. However, the wines were more acidic than the control, and lost part of their fruitiness. Other authors have used encapsulated enzymes in order to improve glucose oxidase activity without prior pH correction [85,86]. Wines obtained from juices treated for two days under these conditions showed a 0.68% (*v*/*v*) reduction in their ethanol content [85].

## 6. Metabolic Inhibitors

Depending on their mode of action, sublethal concentrations of some metabolic inhibitors might be expected to alter the ethanol yield of *S. cerevisiae*. This is the case of furfural, an aromatic aldehyde resulting from sugar dehydration. It can be found in wines at concentrations below the perception limit (20 mg/L). Furfural is toxic for *S. cerevisiae*, and the detoxification mechanism involves its transformation to furfuryl alcohol by alcohol dehydrogenase, the same enzyme catalysing the final step of alcoholic fermentation from acetaldehyde to ethanol. The use of furfural has been proposed to reduce the ethanol yield during wine fermentation [87]. However, the suggested dose, 50 mg/L, is clearly above the perception limit, for a modest reduction in ethanol content of 0.6% (*v*/*v*). The same research group suggested other metabolic inhibitors to be used for the same purpose [88], but no experimental results have been published yet.

## 7. Conclusions

The metabolic versatility of oenological yeasts, considering both non-*Saccharomyces* and *Saccharomyces* species, and our growing knowledge about it, can be exploited to address the problem of the rising alcohol levels observed over the last few decades in wine. Research groups have approached the problem from various perspectives: the exploration of natural genetic diversity, focusing mainly on non-*Saccharomyces* yeasts; the genetic improvement of *S. cerevisiae* by genetic engineering, random mutagenesis, or experimental evolution; or the modification of the environmental conditions (metabolic inhibitors, aerobiosis). A combination of aerobic conditions with yeast genetic improvement (of both *Saccharomyces* and non-*Saccharomyces* yeast strains) is currently one of the most promising options. However, considering the metabolic diversity of wine yeast species and the impact of oxygen on yeast metabolism, any technological improvement developed in the laboratories must be validated by the sensory analysis of the wines produced at the pilot or industrial scale.

## Figures and Tables

**Table 1:** Fermentation conditions and the results obtained for alcohol level reduction using strains of different non-*Saccharomyces* wine yeast species on natural grape must.

Single Species	Sugar Content (g/L)	Conditions	Sensory Analysis ^c^	Time for *S. cerevisiae* Addition	Ethanol Reduction (% ABV)	Reference
*Saccharomyces uvarum*	171 ^a^	standard	Negative	No. Pure culture	0.5	[37]
	240 ^b^	standard	Negative^c^	No. Pure culture	1.7	[38]
	210–240 ^a^	standard	No ^c^	at 50% sugars	0.8–0.9	[39]
*Metschnikowia pulcherrima*	230–240 ^a^	standard	No ^c^	at 50% sugars	0.9–1.6	[40]
	210–240 ^a^	standard	No ^c^	at 50% sugars	1.0–1.1	[39]
	240 ^b^	standard	No different ^c^	0 h (1/10)	1.0	[38]
	230 ^a^	standard	No ^c^	3 days	0.8–1.25	[41]
	264	standard	No different ^c^	3 days	1.0	[42]
	260 ^a^	20 VVH discont 48 h	No	0 h	3.7	[43]
	212 ^a^	DO = 20% 3 days	Negative ^c^	72 h	3.6	[44]
	220 ^a^	3 VVH 72 h	No ^c^	72 h	1.5	[45]
*Starmerella bacillaris*	244	standard	Different	24 h	0.5–0.6	[46]
(*Candida zemplinina*)	250	standard	No	48 h	0.5	[47]
	212 ^a^	DO = 20% 2–3 days	Negative ^c^	69–52 h	0.6	[44]
*Starmerella bombicola*	218	1.2 VVH O_2_ 72 h	No ^c^	72 h	1.5	[48]
*Hanseniaspora uvarum*	230–236	standard	Different ^c^	7 days	0.8–1.1	[49]
	238	standard	Positive ^c^	48 h	0.9	[50]
*Hanseniaspora opuntiae*	230–236	standard	Different ^c^	7 days	0.6–1.3	[49]
*Hanseniaspora vineae*	252 ^a^	20 VVH 24 h	No ^c^	24 h	2.5	[51]
*Torulaspora delbrueckii*	223	standard	Positive ^c^	4 days	0.5	[52]
	195 ^a^	standard	No different ^c^	No. Pure culture	0.5	[53]
	220 ^a^	1.5–3 VVH 72 h	No ^c^	72 h	0.9–1.0	[45]
*Candida oleophila*	206 ^a^	DO = 20% 5 days	Negative ^c^	120 h	1.4	[44]
*Pichia kudriavzevii*	230–236	standard	Different ^c^	7 days	0.4–0.6	[49]
*Pichia guilliermondii*	206 ^a^	DO = 20% 5 days	Negative ^c^	120 h	3.6	[44]
	212 ^a^	DO = 20% 3 days	Negative ^c^	72 h	1.8	[44]
*Pichia kluyveri*	212 ^a^	DO = 20% 3 days	Negative ^c^	72 h	2.7	[44]
*Lachancea thermotolerans*	222	standard	Higher acidity ^c^	48 h	0.7	[54]
	220 ^a^	standard	Negative ^c^	6 days	1.2	[55]
*Meyerozyma guilliermondii*	230 ^a^	standard	No ^c^	3 days	1.2	[41]
*Zygosaccharomyces bailii*	220 ^a^	3 VVH 72 h	No ^c^	72 h	1.2	[45]
**Two Species**						
*M. pulcherrima/S. uvarum*	210–240 ^a^	standard	No ^c^	at 50% sugars	1.8	[39]
*M. pulcherrima/S. bayanus*	195 ^a^	standard	Positive ^c^	96 h	0.9	[53]
**Immobilized**						
*Hanseniaspora osmophila*	202	standard	No ^c^	72 h	1.0	[56]
*Hanseniaspora uvarum*	202	standard	No ^c^	72 h	1.2	[56]
*Starmerella bombicola*	202	standard	No ^c^	72 h	1.6	[56]
*Metschnikowia pulcherrima*	202	standard	No ^c^	72 h	1.5	[56]
	204 ^a^	1.2 VVH 72 h	No ^c^	72 h	1.4	[57]
*Schizosaccharomyces japonicus*	240 ^a^	standard	No ^c^	0 h/48 h	1.7/2.4	[58]

^a^ Heat treated or filter sterilized; ^b^ DMDC treated; ^c^ Volatile Compound Analysis performed; DO: dissolved oxygen; VVH: volume per volume per hour.

## Data Availability

Not applicable.

## References

[1] Mira de Orduña R. (2010). Climate change associated effects on grape and wine quality and production. Food Res. Int..

[2] Goold H.D., Kroukamp H., Williams T.C., Paulsen I.T., Varela C., Pretorius I.S. (2017). Yeast’s balancing act between ethanol and glycerol production in low-alcohol wines. Microb. Biotechnol..

[3] Palacios A., Raginel F., Ortiz-Julien A. (2007). Can the selection *Saccharomyces cerevisiae* yeast lead to variations in the final alcohol degree of wines?. Aust. N. Zeal. Grapegrow. Winemak..

[4] Camarasa C., Sanchez I., Brial P., Bigey F., Dequin S. (2011). Phenotypic landscape of *Saccharomyces cerevisiae* during wine fermentation: Evidence for origin-dependent metabolic traits. PLoS ONE.

[5] Cebollero E., González-Ramos D., Tabera L., Gonzalez R. (2007). Transgenic wine yeast technology comes of age: Is it time for transgenic wine?. Biotechnol. Lett..

[6] Gonzalez R., Tronchoni J., Quirós M., Morales P., Moreno-Arribas M.V., Bartolomé Sualdea B. (2016). Genetic improvement and genetically modified microorganisms. Wine Safety, Consumer Preference, and Human Health.

[7] Drewke C., Thielen J., Ciriacy M. (1990). Ethanol formation in adh0 mutants reveals the existence of a novel acetaldehyde-reducing activity in *Saccharomyces cerevisiae*. J. Bacteriol..

[8] Dequin S., Barre P. (1994). Mixed lactic acid-alcoholic fermentation by *Saccharomyces cerevisiae* expressing the Lactobacillus casei L(+)-LDH. Nat. Biotechnol..

[9] Michnick S., Roustan J.L., Remize F., Barre P., Dequin S. (1997). Modulation of glycerol and ethanol yields during alcoholic fermentation in *Saccharomyces cerevisiae* strains overexpressed or disrupted for GPD1 encoding glycerol 3-phosphate dehydrogenase. Yeast.

[10] Remize F., Andrieu E., Dequin S. (2000). Engineering of the pyruvate dehydrogenase bypass in *Saccharomyces cerevisiae*: Role of the cytosolic Mg2+ and mitochondrial K+ acetaldehyde dehydrogenases Ald6p and Ald4p in acetate formation during alcoholic fermentation. Appl. Environ. Microbiol..

[11] Cambon B., Monteil V., Remize F., Camarasa C., Dequin S. (2006). Effects of GPD1 overexpression in *Saccharomyces cerevisiae* commercial wine yeast strains lacking ALD6 genes. Appl. Environ. Microbiol..

[12] Ehsani M., Fernández M.R., Biosca J.A., Julien A., Dequin S. (2009). Engineering of 2,3-butanediol dehydrogenase to reduce acetoin formation by glycerol-overproducing, low-alcohol *Saccharomyces cerevisiae*. Appl. Environ. Microbiol..

[13] Heux S., Sablayrolles J.-M., Cachon R., Dequin S. (2006). Engineering a *Saccharomyces cerevisiae* wine yeast that exhibits reduced ethanol production during fermentation under controlled microoxygenation conditions. Appl. Environ. Microbiol..

[14] Flikweert M.T., van der Zanden L., Janssen W.M.T.M., Steensma H.Y., van Dijken J.P., Pronk J.T. (1996). Pyruvate decarboxylase: An indispensable enzyme for growth of *Saccharomyces cerevisiae* on glucose. Yeast.

[15] Nevoigt E., Stahl U. (1996). Reduced pyruvate decarboxylase and increased glycerol-3-phosphate dehydrogenase [NAD+] levels enhance glycerol production in *Saccharomyces cerevisiae*. Yeast.

[16] Cuello R.A., Montero K.J.F., Mercado L.A., Combina M., Ciklic I.F. (2017). Construction of low-ethanol–wine yeasts through partial deletion of the *Saccharomyces cerevisiae* PDC2 gene. AMB Expr..

[17] Dai Z., Huang M., Chen Y., Siewers V., Nielsen J. (2018). Global rewiring of cellular metabolism renders *Saccharomyces cerevisiae* Crabtree negative. Nat. Commun..

[18] Otterstedt K., Larsson C., Bill R.M., Stahlberg A., Boles E., Hohmann S., Gustafsson L. (2004). Switching the mode of metabolism in the yeast *Saccharomyces cerevisiae*. EMBO Rep..

[19] Compagno C., Brambilla L., Capitanio D., Boschi F., Ranzi B.M., Porro D. (2001). Alterations of the glucose metabolism in a triose phosphate isomerase-negative *Saccharomyces cerevisiae* mutant. Yeast.

[20] Cordier H., Mendes F., Vasconcelos I., Francois J.M. (2007). A metabolic and genomic study of engineered *Saccharomyces cerevisiae* strains for high glycerol production. Metab. Eng..

[21] Rossouw D., Heyns E.H., Setati M.E., Bosch S., Bauer F.F. (2013). Adjustment of trehalose metabolism in wine *Saccharomyces cerevisiae* strains to modify ethanol yields. Appl. Environ. Microbiol..

[22] Du Q., Liu Y., Song Y., Qin Y. (2020). Creation of a low-alcohol-production yeast by a mutated SPT15 transcription regulator triggers transcriptional and metabolic changes during wine fermentation. Front. Microbiol..

[23] Malherbe D.F., du Toit M., Cordero Otero R.R., van Rensburg P., Pretorius I.S. (2003). Expression of the *Aspergillus niger* glucose oxidase gene in *Saccharomyces cerevisiae* and its potential applications in wine production. Appl. Microbiol. Biotechnol..

[24] Mans R., van Rossum H.M., Wijsman M., Backx A., Kuijpers N.G., van den Broek M., Daran-Lapujade P., Pronk J., van Maris A.J.A., Daran J.M.G. (2015). CRISPR/Cas9: A molecular Swiss army knife for simultaneous introduction of multiple genetic modifications in *Saccharomyces cerevisiae*. FEMS Yeast Res..

[25] Vigentini I., Gebbia M., Belotti A., Foschino R., Roth F.P. (2017). CRISPR/Cas9 system as a valuable genome editing tool for wine yeasts with application to decrease urea production. Front. Microbiol..

[26] Van Wyk N., Kroukamp H., Espinosa M.I., von Wallbrunn C., Wendland J., Pretorius I.S. (2020). Blending wine yeast phenotypes with the aid of CRISPR DNA editing technologies. Int. J. Food Microbiol..

[27] Gonzalez R., Morales P. (2021). Truth in wine yeast. Microb. Biotechnol..

[28] Cadière A., Ortiz-Julien A., Camarasa C., Dequin S. (2011). Evolutionary engineered *Saccharomyces cerevisiae* wine yeast strains with increased in vivo flux through the pentose phosphate pathway. Metab. Eng..

[29] Cadière A., Aguera E., Caillé S., Ortiz-Julien A., Dequin S. (2012). Pilot-scale evaluation the enological traits of a novel, aromatic wine yeast strain obtained by adaptive evolution. Food Microbiol..

[30] Taherzadeh M.J., Adler L., Liden G. (2002). Strategies for enhancing fermentative production of glycerol—A review. Enzym. Microb. Technol..

[31] Kutyna D.R., Varela C., Stanley G.A., Borneman A.R., Henschke P.A., Chambers P.J. (2012). Adaptive evolution of *Saccharomyces cerevisiae* to generate strains with enhanced glycerol production. Appl. Microbiol. Biotechnol..

[32] Blomberg A., Adler L. (1992). Physiology of osmotolerance in fungi. Adv. Microb. Physiol..

[33] Tilloy V., Ortiz-Julien A., Dequin S. (2014). Reduction of ethanol yield and improvement of glycerol formation by adaptive evolution of the wine yeast *Saccharomyces cerevisiae* under hyperosmotic conditions. Appl. Environ. Microbiol..

[34] Mas A., Guillamón J.M., Beltran G. (2016). Editorial: Non-conventional yeast in the wine industry. Front. Microbiol..

[35] Vejarano R., Gil-Calderón A. (2021). Commercially available non-*Saccharomyces* yeasts for winemaking: Current market, advantages over *Saccharomyces*, biocompatibility, and safety. Fermentation.

[36] Ciani M., Morales P., Comitini F., Tronchoni J., Canonico L., Curiel J.A., Oro L., Rodrigues A.J., Gonzalez R. (2016). Non-conventional yeast species for lowering ethanol content of wines. Front. Microbiol..

[37] Coloretti F., Zambonelli C., Tini V. (2006). Characterization of flocculent *Saccharomyces* interspecific hybrids for the production of sparkling wines. Food Microbiol..

[38] Varela C., Barker A., Tran T., Borneman A., Curtin C. (2017). Sensory profile and volatile aroma composition of reduced alcohol Merlot wines fermented with *Metschnikowia pulcherrima* and *Saccharomyces Uvarum*. Int. J. Food Microbiol..

[39] Varela C., Sengler F., Solomon M., Curtin C. (2016). Volatile flavour profile of reduced alcohol wines fermented with the non-conventional yeast species *Metschnikowia pulcherrima* and *Saccharomyces Uvarum*. Food Chem..

[40] Contreras A., Hidalgo C., Schmidt S., Henschke P.A., Curtin C., Varela C. (2014). Evaluation of non-*Saccharomyces* yeasts for the reduction of alcohol content in wine. Appl. Environ. Microbiol..

[41] García M., Esteve-Zarzoso B., Cabellos J.M., Arroyo T. (2020). Sequential *non-Saccharomyces* and *Saccharomyces cerevisiae* fermentations to reduce the alcohol content in wine. Fermentation.

[42] Aplin J.J., Paup V.D., Ross C.F., Edwards C.G. (2021). Chemical and sensory profiles of Merlot wines produced by sequential inoculation of *Metschnikowia pulcherrima* or *Meyerozyma guilliermondii*. Fermentation.

[43] Morales P., Rojas V., Quirós M., Gonzalez R. (2015). The impact of oxygen on the final alcohol content of wine fermented by a mixed starter culture. Appl. Microbiol. Biotechnol..

[44] Röcker J., Strub S., Ebert K., Grossmann M. (2016). Usage of different aerobic non-*Saccharomyces* yeasts and experimental conditions as a tool for reducing the potential ethanol content in wines. Eur. Food Res. Technol..

[45] Canonico L., Solomon M., Comitini F., Ciani M., Varela C. (2019). Volatile profile of reduced alcohol wines fermented with selected non-*Saccharomyces* yeasts under different aeration conditions. Food Microbiol..

[46] Giaramida P., Ponticello G., Di Maio S., Squadrito M., Genna G., Barone E., Scacco A., Amore G., di Stefano R., Oliva D. (2013). *Candida zemplinina* for production of wines with less alcohol and more glycerol. S. Afr. J. Enol. Vitic..

[47] Englezos V., Rantsiou K., Cravero F., Torchio F., Ortiz-Julien A., Gerbi V., Rolle L., Cocolin L. (2016). *Starmerella bacillaris* and *Saccharomyces cerevisiae* mixed fermentations to reduce ethanol content in wine. Appl. Microbiol. Biotechnol..

[48] Canonico L., Galli E., Agarbati A., Comitini F., Ciani M. (2021). *Starmerella bombicola* and *Saccharomyces cerevisiae* in wine sequential fermentation in aeration condition: Evaluation of ethanol reduction and analytical profile. Foods.

[49] Rossouw D., Bauer F.F. (2016). Exploring the phenotypic space of non-*Saccharomyces* wine yeast biodiversity. Food Microbiol..

[50] Mestre M.V., Maturano Y.P., Gallardo C., Combina M., Mercado L., Toro M.E., Carrau F., Vazquez F., Dellacassa E. (2019). Impact on sensory and aromatic profile of low ethanol Malbec wines fermented by sequential culture of *Hanseniaspora uvarum* and *Saccharomyces cerevisiae* native yeasts. Fermentation.

[51] Yan G., Zhang B., Joseph L., Waterhouse A.L. (2020). Effects of initial oxygenation on chemical and aromatic composition of wine in mixed starters of *Hanseniaspora vineae* and *Saccharomyces cerevisiae*. Food Microbiol..

[52] Belda I., Ruiz J., Beisert B., Navascués E., Marquina D., Calderón F., Rauhut D., Benito S., Santos A. (2017). Influence of *Torulaspora delbrueckii* in varietal thiol (3-SH and 4-MSP) release in wine sequential fermentations. (2018). Int. J. Food Microbiol..

[53] Puškaš V.S., Miljić U.D., Djuran J.J., Vučurović M.M. (2019). The aptitude of commercial yeast strains for lowering the ethanol content of wine. Food Sci. Nutr..

[54] Gobbi M., Comitini F., Domizio P., Romani C., Lencioni L., Mannazzu I., Ciani M. (2013). *Lachancea thermotolerans* and *Saccharomyces cerevisiae* in simultaneous and sequential co-fermentation: A strategy to enhance acidity and improve the overall quality of wine. Food Microbiol..

[55] Del Fresno J.M., Morata A., Loira I., Bañuelos M.A., Escott C., ·Benito S., González Chamorro C., Suárez-Lepe J.A. (2017). Use of non-*Saccharomyces* in single-culture, mixed and sequential fermentation to improve red wine quality. Eur. Food Res. Technol..

[56] Canonico L., Comitini F., Oro L., Ciani M. (2016). Sequential fermentation with selected immobilized non-*Saccharomyces* yeast for reduction of ethanol content in wine. Front. Microbiol..

[57] Canonico L., Comitini F., Ciani M. (2019). *Metschnikowia pulcherrima* selected strain for ethanol reduction in wine: Influence of cell immobilization and aeration condition. Foods.

[58] Domizio P., Lencioni L., Calamai L., Portaro L., Bisson L.F. (2018). Evaluation of the yeast *Schizosaccharomyces japonicus* for use in wine production. Am. J. Enol. Vitic..

[59] Castellari L., Ferruzzi M., Magrini A., Giudici P., Passarelli P., Zambonelli C. (1994). Unbalanced wine fermentation by cryotolerant vs. Non cryotolerant *Saccharomyces* strains. Vitis.

[60] Giudici P., Zambonelli C., Passarelli P., Castellari L. (1995). Improvement of wine composition with cryotolerant *Saccharomyces* strains. Am. J. Enol. Vitic..

[61] Arroyo-Lopez F.N., Perez-Torrado R., Querol A., Barrio E. (2010). Modulation of the glycerol and ethanol syntheses in the yeast *Saccharomyces kudriavzevii* differs from that exhibited by *Saccharomyces cerevisiae* and their hybrid. Food Microbiol..

[62] Gangl H., Batusic M., Tscheik G., Tiefenbrunner W., Hack C., Lopandic K. (2009). Exceptional fermentation characteristics of natural hybrids from *Saccharomyces cerevisiae* and S. *kudriavzevii*. New Biotechnol..

[63] Magyar I., Toth T. (2011). Comparative evaluation of some oenological properties in wine strains of *Candida stellata*, *Candida zemplinina*, *Saccharomyces uvarum* and *Saccharomyces cerevisiae*. Food Microbiol..

[64] Di Maio S., Genna G., Gandolfo V., Amore G., Ciaccio M., Oliva D. (2012). Presence of *Candida zemplinina* in Sicilian musts and selection of a strain for wine mixed fermentations. S. Afr. J. Enol. Vitic..

[65] Englezos V., Rantsiou K., Torchio F., Rolle L., Gerbi V., Cocolin L. (2015). Exploitation of the non-*Saccharomyces* yeast *Starmerella bacillaris* (synonym *Candida zemplinina*) in wine fermentation: Physiological and molecular characterizations. Int. J. Food Microbiol..

[66] Gobbi M., De Vero L., Solieri L., Comitini F., Oro L., Giudici P., Ciani M. (2014). Fermentative aptitude of non-*Saccharomyces* wine yeast for reduction in the ethanol content in wine. Eur. Food Res. Technol..

[67] Mestre M.V., Maturano Y.P., Combina M., Mercado L.A., Toro M.E., Vazquez F. (2017). Selection of non-*Saccharomyces* yeasts to be used in grape musts with high alcoholic potential: A strategy to obtain wines with reduced ethanol content. FEMS Yeast Res..

[68] Morata A., Loira I., Tesfaye W., Bañuelos M.A., González C., Suárez Lepe J.A. (2018). *Lachancea thermotolerans* applications in wine technology. Fermentation.

[69] Erten H., Campbell I. (2001). The production of low-alcohol wines by aerobic yeasts. J. Inst. Brew..

[70] Gonzalez R., Quirós M., Morales P. (2013). Yeast respiration of sugars by non-*Saccharomyces* yeast species: A promising and barely explored approach to lowering alcohol content of wines. Trends Food Sci. Technol..

[71] Quirós M., Rojas V., Gonzalez R., Morales P. (2014). Selection of non-*Saccharomyces* yeast strains for reducing alcohol levels in wine by sugar respiration. Int. J. Food Microbiol..

[72] Rodrigues A.J., Raimbaud T., Gonzalez R., Morales P. (2016). Environmental factors influencing the efficacy of different yeast strains for alcohol level reduction in wine by respiration. LWT Food Sci. Technol..

[73] Tronchoni J., Curiel J.A., Sáenz-Navajas M.P., Morales P., de-la-Fuente-Blanco A., Fernández-Zurbano P., Ferreira V., Gonzalez R. (2018). Aroma profiling of an aerated fermentation of natural grape must with selected yeast strains at pilot scale. Food Microbiol..

[74] Contreras A., Hidalgo C., Schmidt S., Henschke P.A., Curtin C., Varela C. (2015). The application of non-*Saccharomyces* yeast in fermentations with limited aeration as a strategy for the production of wine with reduced alcohol content. Int. J. Food Microbiol..

[75] Curiel J.A., Salvadó Z., Tronchoni J., Morales P., Rodrigues A.J., Quirós M., Gonzalez R. (2016). Identification of target genes to control acetate yield during aerobic fermentation with *Saccharomyces cerevisiae*. Microb. Cell Fact..

[76] Guindal A.M., Morales P., Gonzalez R., Tronchoni J. Adaptive Laboratory Evolution to reduce acetic acid yield of *Saccharomyces cerevisiae* wine yeast strains under aerobic conditions. Proceedings of the 30th International Conference on Yeast Genetics and Molecular Biology (Virtual Conference).

[77] Tronchoni J., Gonzalez R., Guindal A.M., Calleja E., Morales P. (2022). Exploring the suitability of *Saccharomyces cerevisiae* strains for winemaking under aerobic conditions. Food Microbiol..

[78] Villettaz J.-C. (1986). Method for Production of a Low Alcoholic Wine and Agent for Performance of the Method. European Patent.

[79] Pickering G.J. (2000). Low- and Reduced-alcohol Wine: A Review. J. Wine Res..

[80] International Code of Oenological Practices. https://www.oiv.int/en/technical-standards-and-documents/oenological-practices/international-code-of-oenological-practices.

[81] Pickering G.J., Heatherbell D.A., Barnes M.F. (1999). Optimising glucose conversion in the production of reduced alcohol wines from glucose oxidase treated musts. Food Res. Int..

[82] Pickering G.J., Heatherbell D.A., Barnes M.F. (1999). The production of reduced-alcohol wine using glucose oxidase treated juice. Part I. Composition. Am. J. Enol. Vitic..

[83] Pickering G.J., Heatherbell D.A., Barnes M.F. (1999). The production of reduced-alcohol wine using glucose oxidase-treated juice. Part III. Sensory. Am. J. Enol. Vitic..

[84] Röcker J., Schmitt M., Pasch L., Ebert K., Grossmann M. (2016). The use of glucose oxidase and catalase for the enzymatic reduction of the potential ethanol content in wine. Food Chem..

[85] Biyela B.N.E., du Toit W.J., Divol B., Malherbe D.F., Van Rensburg P. (2009). The production of reduced-alcohol wines using Gluzyme Mono 10.000 BG-treated grape juice. S. Afr. J. Enol. Vitic..

[86] Ruiz E., Busto M.D., Ramos-Gómez S., Palacios D., Pilar-Izquierdo M.C., Ortega N. (2018). Encapsulation of glucose oxidase in alginate hollow beads to reduce the fermentable sugars in simulated musts. Food Biosci..

[87] Abalos D., Vejarano R., Morata A., González C., Suárez-Lepe J.A. (2001). The use of furfural as a metabolic inhibitor for reducing the alcohol content of model wines. Eur. Food Res. Technol..

[88] Vejarano R., Morata A., Loira I., González M.C., Suárez-Lepe J.A. (2013). Theoretical considerations about usage of metabolic inhibitors as possible alternative to reduce alcohol content of wines from hot areas. Eur. Food Res. Technol..

